# Specific and common therapeutic factors in psychodynamic psychotherapy for children and adolescents: an overview

**DOI:** 10.3389/fpsyg.2025.1525849

**Published:** 2025-01-23

**Authors:** Sabine Sammer-Schreckenthaler, Gloria Lagetto, Human-Friedrich Unterrainer, Omar C. G. Gelo

**Affiliations:** ^1^Faculty of Psychotherapy Science, Sigmund Freud University, Vienna, Austria; ^2^Department of Psychology and Educational Sciences, Pegaso Telematic University, Naples, Italy; ^3^Department of Religious Studies, University of Vienna, Vienna, Austria; ^4^University Clinic for Psychiatry and Psychotherapeutic Medicine, Medical University of Graz, Graz, Austria; ^5^Center for Integrative Addiction Research (CIAR), Grüner Kreis Society, Vienna, Austria; ^6^Department of Human and Social Sciences, University of Salento, Lecce, Italy

**Keywords:** psychodynamic psychotherapy, children, adolescents, common factors, specific factors

## Abstract

The effectiveness of psychodynamic psychotherapy for children and adolescents (PPCA) has been increasingly demonstrated by a growing number of meta-analyses. However, very little is still known about the therapeutic factors responsible for this effectiveness. On the one hand, some authors have suggested that PPCA works because of specific therapeutic factors. On the other hand, it has been suggested that the effectiveness of PPCA may be due to factors common to different approaches. In the present paper, we provide an overview and discuss some of the existing clinical-theoretical and empirical literature on specific and common factors of PPCA. Several specific and common factors of PPCA were identified. Regarding the former, these included clinical processes (insight; working through; remembering and reconstructing; catharsis, abreaction, and regression; and transference and countertransference) and therapeutic techniques (interpretation of transference, countertransference, dreams, defense mechanisms, and resistance; verbalization; mirroring; and free play). Regarding the latter, these included relational factors (therapeutic alliance and interaction structures), patient factors (willingness to participate, readiness for change, treatment involvement, and positive expectations and hope), therapist factors (interpersonal skills, direct influence skills, credibility, involving parents, playing ability, flexibility, and allegiance), parent and interpersonal environment factors (parental willingness to participate, treatment involvement, treatment expectations, and perceived barriers to treatment participation and therapeutic change; family dynamics; parent-therapist alliance; and social support), mentalizing (of the therapist, client, and parents), and play (symbolization, affect regulation, mental state talk, and patterns of interaction). PPCA appears to work through both specific and common factors, more likely through their synergic interaction. However, empirical support for these therapeutic factors and their mutual interaction remains sparse. Future qualitative and quantitative research should address more in detail the extent to which specific factors, common factors, or both account for the effectiveness of PPCA. Identifying empirically supported specific and common factors and their possible interaction can inform and improve clinical practice and training.

## 1 Introduction

How and why psychotherapy works represents a fundamental question in psychotherapy science (Gelo et al., 2015a). At least from a certain perspective, this question refers to the identification of so-called therapeutic factors, which represent aspects of the therapeutic process responsible for patient change. It has been suggested that therapeutic factors may be either *specific* to a particular treatment approach or *common* to several approaches (McAleavey and Castonguay, 2015; Wampold and Imel, 2015). However, despite the progress made in the field, much remains to be done conceptually and empirically to clarify better the role of specific and common factors in psychotherapy in general and in each particular school of psychotherapy. This is especially the case for psychodynamic psychotherapy for children and adolescents (PPCA).

Psychodynamic psychotherapy has a long history in the treatment of children and adolescents. PPCA encompasses a range of approaches (Delgado et al., 2015) that nonetheless share a set of basic assumptions, such as the unconscious and affect-laden nature of mental processes, their influence on our behavior and relationships (both in everyday life and in the therapeutic relationship), the importance of transference and countertransference in the therapeutic relationship, and the overall goal of fostering clients’ self-awareness, self-understanding, and reflective abilities along with the development of broader and more flexible relational patterns (Kernberg et al., 2012). These assumptions have been reflected in various theories of change addressing the question of how and why PPCA works and revolving around some basic therapeutic factors. Some of these factors can be considered specific to PPCA. For example, in more classical approaches, facilitating the child’s and adolescent’s expression of symbolic material (behavior, verbal expressions, and free play), its interpretation within a transferential/countertransferential relationship, and the resulting emotional insight (or the analysis of transference/countertransference). In more recent approaches, fostering an intersubjective field in the here-and-now (e.g., through mirroring techniques) within which implicit relational memories can be reorganized and mentalizing abilities may be enhanced so that corrective emotional experiences take place (Delgado et al., 2015; Hayes and Brunst, 2017). At the same time, it has been suggested that some other therapeutic factors of PPCA are common to other schools of psychotherapy for children and adolescents [e.g., humanistic (Ray and Jayne, 2016), cognitive-behavioral (Cornacchio et al., 2017), systemic (Huang et al., 2024); see also Porter et al., 2009]. Examples are the children and adolescents’ expectations, readiness for change, and involvement; the therapist’s interpersonal skills and ability to provide a clear and well-defined setting; and the child/adolescent-therapist alliance as well as the parent-therapist alliance (Hayes, 2017; Karver et al., 2005).

Although the effectiveness of PPCA has been demonstrated to some extent for a variety of populations and diagnoses (e.g., Abbass et al., 2013; Kronmüller et al., 2010; Midgley et al., 2017), the role that specific and common factors play in PPCA remains unclear (see Kazdin, 2002). This seems to be due to two main reasons. First, the clinical-theoretical literature on PPCA (as in any therapeutic approach) tends to emphasize the role of specific factors. This should not be surprising, given the identity role that specific factors play for a particular school of psychotherapy in terms of its specific theory of change. However, this seems to have come at the expense of an explicit reflection on the role of common factors in PPCA. Second, empirical research on specific and/or common therapeutic factors in PPCA is still sparse. If this is true for research on specific factors, it is even more true for research on common factors (for a review, see Karver et al., 2005; Midgley, 2007; Ng et al., 2021; Hayes, 2017; Hayes and Brunst, 2017; Shirk and Burwell, 2010). As stated by Hayes (2017), “research into common factors in youth therapy is still in his infancy, with some areas embryonic or maybe even just a twinkle in the eye of a therapy researcher.” (p. 141).

In this paper, we want to take stock of both specific and common factors in PPCA with regard to clinical-theoretical and empirical literature. A comprehensive and systematic literature review is beyond the scope of this article. Rather, we intend to describe and discuss those specific and common factors we consider mostly relevant in PPCA. In doing so, we will refer to the clinical-theoretical literature addressing these factors and the empirical findings providing (at least an initial) support for them. We conclude with some recommendations for research, practice, and training.

## 2 Specific factors in PPCA

In discussing the specific factors of PPCA, we distinguish between *clinical processes* and *therapeutic techniques* (see Table 1). The former are the clinically desirable processes required for treatment progress. The latter are specific actions and practices (e.g., conversational moves, behaviors, and activities) that the therapist implements to favor the change process in therapy (Hayes and Brunst, 2017).

**Table 1:** Specific factors of PPCA.

Domain	Therapeutic factor
Clinical processes	Insight
	Working through
	Remembering and reconstructing
	Catharsis, abreaction, and regression
	Transference and countertransference
Therapeutic techniques	Interpretation (of transference and countertransference, dreams, defense mechanisms, and resistance)
	Verbalization
	Mirroring
	Free play

### 2.1 Clinical processes

#### 2.1.1 Insight

Insight is a fundamental clinical process in PPCA. In classical one-person approaches, it is described as an increased awareness of unconscious wishes and conflicts and is considered to occur “through recognition of maladaptive ego defenses, the presence of transference manifestations (i.e., remembering and repeating), or by discovering object relations conflicts, which are amenable for being worked through by verbal insight-oriented suggestions or interpretations” (Delgado et al., 2015, p. 40). Following such a traditional view, insight is mainly due to the therapist’s interpretation. Importantly, it is assumed that the possibility of promoting insight (i.e., making unconscious impulses and wishes conscious) and defending against them requires children and adolescents who have already reached a certain level of symbolic ability (Göttken and von Klitzing, 2015). In more recent two-person PPCA, insight has been reconceptualized in more general terms regarding the concept of mentalization—defined as the ability to understand one’s own and other’s mental states (Fonagy and Allison, 2014)—and, as such, it is seen to result from corrective emotional experiences (Castonguay and Hill, 2012) (see a later section in this article).

In the treatment of adults, some empirical studies dealing with the role of insight can be found (e.g., Johansson et al., 2010; Leichsenring and Leibing, 2007), with some reviews and meta-analyses indicating that insight contributes to an effective therapeutic action (e.g., Lacewing, 2014; Jennissen et al., 2018). In contrast, there appear to be no empirical studies in child psychotherapy that explicitly address the role of insight in the change process of PPCA.

#### 2.1.2 Working through

Strictly connected to insight is the process of working through, consisting of the client “recognizing resistances (insight) and overcoming resistances (change)” (Sedler, 1983, p. 73) in a process where the increased awareness (insight) resulting from the therapist’s interpretation over the treatment is assimilated, incorporated, and integrated into the patient’s psychic life (LaLonde and Dauphin, 2017). The process of working through is referred to in PPCA’s clinical literature (e.g., LaLonde and Dauphin, 2017), case studies (e.g., Trad et al., 1992), and treatment manuals (e.g., Normandin et al., 2015), but empirical studies specifically focusing on it are lacking. For example, in the context of Transference-Focused Psychotherapy for borderline adolescents, it has been stated that analogously to adult treatment, working through plays a relevant role, although it is to be expected at a slower rate (Normandin et al., 2015). Moreover, two studies on the effectiveness of PPCA used manuals in which working through was one of three treatment phases, along with getting to know each other and saying goodbye (Horn et al., 2005; Kronmüller et al., 2005). Finally, Delgado and Strawn (2012) wrote about the duration of the termination phase of psychoanalytic psychotherapy with adolescents and the relevance of working through, especially in the final phases of the treatment.

#### 2.1.3 Remembering and reconstructing

Remembering and reconstructing are processes through which process can be set in motion, with interpretations (see a later section in this paper) promoting the process of reconstruction and integration for a coherent self-experience. Fragmented and repressed memories can thus be remembered, retrieved, and processed. Both remembering and reconstructing have lost importance since the concept of intersubjectivity has come to the fore within contemporary approaches (Blum, 2005). Remembering and reconstructing can be problematic with children and adolescents because of their developmental stage. For this reason, reconstruction often occurs with the parents or caregivers, referring to pre-, peri-, and postnatal events and (early) child development. It also takes place in the context of initial or anamnesis interviews. The situation is different for adolescent patients. Especially in biographies characterized by many relationship breakdowns, foster families, or institutional placement, the reconstruction of one’s life path is very important.

In a case vignette, Pretorius (2007) describes the role of remembering and repeating in the treatment of a six-year-old boy who suffered from an early trauma. Remembering plays an important role in the treatment of (early) trauma (Alvarez, 1992; Gaensbauer, 2002). Diatkine (1993) addressed the question of the extent to which reconstruction is meaningful and possible in the psychoanalytic treatment of children. After all, the psychoanalyst depends on caregivers’ narratives or direct observations. The question arises as to whether a congruent reconstruction is the goal of psychoanalytic treatment or whether it is more about the personal narrative of a child. Prot (2010) describes a case vignette based on an important child reconstruction that positively influenced the patient’s psychotherapeutic change. Finally, Brainin (2009) describes challenges in treating adolescents concerning reconstruction and notes that these are sometimes perceived as threatening by adolescents as they are in the process of detaching from the primary family. Notwithstanding these criticalities, reconstruction can be a relevant factor in PPCA, including in the treatment of children with post-traumatic stress disorder. For example, early trauma (experienced around 28–36 months) is more likely to manifest itself through memories at a behavioral level than at a verbal level, and reconstructive techniques can be a helpful approach also in such cases (Terr, 1989).

The clinical relevance of reconstruction with children has been acknowledged by Muratori et al. (2003). In a follow-up study conducted 2 years after receiving short-term psychodynamic psychotherapy, the focus of the manual in the first five sessions was, among others, on reconstruction.

#### 2.1.4 Catharsis, abreaction and regression

In their descriptions of the most important elements of therapeutic play, Schaefer and Drewes (2013) refer to catharsis, abreaction, and regression as core elements of play (see a later section in this paper). In PPCA, catharsis and abreaction relate to the “flow experience” achieved through play (see Levy, 2008; Schaefer and Drewes, 2013). Since play is an action, it can also lead to an abreaction on the action level or the possibility of emotional release. Although both terms may be considered historically rather outdated concepts, they are still relevant in some approaches to PPCA. For example, Lehmhaus and Reiffen-Züger (2018) hold that catharsis is in play, and the associated abreaction plays a role in PPCA (see also Levenson and Herman, 1991). Analogously, Terradas and Asselin (2021) ascribe a specific abreactive function to play in their four-stage play model. However, it should be considered that the relevance of these concepts is decreasing along the evolution of PPCA from a one-person to a two-person psychology.

Regarding regression in PPCA, much has been written on the psychodynamic view of regression, its different types and manifestations, or potentials and dangers (Freud, 2018; Wiese, 1983; Winnicott, 2010; Silverman, 1985). At a very general level, regression can potentially have an ego-strengthening and integrating effect. At the same time, it should be reminded that children and adolescents with ego-structural deficits need to be protected from a “free fall” into regression by the therapists through support. In contrast, in neurotic children, regression can promote a clinical process that leads to change. We are not aware of any empirical studies investigating catharsis, abreaction, or regression in PPCA.

#### 2.1.5 Transference and countertransference

Transference and countertransference refer to the fact that the unconscious processes of the patient and therapist (whether unconscious fantasies displaced onto the other or mutually enacted implicit patterns of relational knowing) affect their relationship (Delgado et al., 2015). In the more classical approaches to PPCA, an abstinent, opaque, and neutral therapist can facilitate the development of the patient’s transference, which, if properly interpreted, promotes therapeutic progress. It follows that the therapist’s countertransference can interfere with the patient’s transference and negatively affect the change process. In contrast, more recent approaches to PPCA emphasize how both patient and therapist enact their own learned patterns of interaction within the relationship. If sufficiently mentalized and analyzed by the therapist, these can enable the patient to experience a more adaptive and functional relationship in the here and now of the therapeutic encounter, benefiting the patient’s relational patterns and therapeutic change.

Given the specificity of PPCA, Gabel and Bemporad (1994) argued for an expansion of the concept of countertransference, especially in work with children when parents are involved: A therapist’s countertransference to the child could be shifted to the parent(s) and, conversely, a therapist’s countertransference to the parent could be acted out on the child. It is, therefore, useful to address the countertransferential relationship with parents or caregivers to facilitate a successful change process.

While transference and countertransference have been empirically investigated to a certain extent in adult psychodynamic psychotherapy (e.g., Marmarosh and Kivlighan, 2012; Rossberg et al., 2007), the same cannot be said for PPCA (Karver et al., 2005). Regarding transference, Luborsky et al. (1996) developed a reliable instrument for assessing transference patterns in children, the Core Conflictual Relationship Theme (CCRT)—Child Version. Unfortunately, no studies are known to the authors employing such an instrument in actual PPCA psychotherapy sessions.

Regarding countertransference, in addition to case studies (e.g., Berlin, 2002), some research has analyzed the therapists’ unconscious interpersonal patterns using the CCRT, showing, for example, that therapists either repeated or repaired the responses they received from their parents in the interaction with the client, and either identified with or withdrew from patients whose relational patterns with their parents was similar to the therapists’ relational patterns with their parents (Tishby and Vered, 2011).

Some studies on psychodynamic adolescent psychotherapy investigated therapists’ emotional reactions toward patients with different versions of the Feeling Word Checklist-24 (FWC; Dahl et al., 2012; Hoffart and Friis, 2000; Holmqvist and Armelius, 1994). Results identified distinct clusters of positive (e.g., confident, motherly, warm, parental, relaxed, playful, or free) and negative (e.g., detached, disengaged, negative, or inadequate) countertransference feelings. In addition, positive and negative countertransference feelings have shown, respectively, positive and negative correlations with therapeutic alliance, mostly when rated by the therapist (Brøsholen et al., 2022; Dahl et al., 2012; Odhammar et al., 2019; Ulberg et al., 2013) and, to a lesser extent, when rated by the client (Brøsholen et al., 2022; Dahl et al., 2012; see also Ness et al., 2018). Finally, countertransference feelings have shown a stronger association with clients’ social functioning than with their symptoms or overall level of functioning (Brøsholen et al., 2022). Interestingly, some studies have found that countertransference feelings are not typical only of psychodynamic psychotherapy but can also be observed in other theoretical orientations (Betan et al., 2005; Ulberg et al., 2013), with positive countertransference feelings being associated with clinician’s practical experience, age, and amount of clinical supervision (Ulberg et al., 2013).

Such a view is supported by other empirical studies that using the Countertransference Questionnaire for Adolescents (CQ-A; Zittel and Westen, 2003) and the Therapist Response Questionnaire for Adolescents (TRQ; Betan et al., 2005; Satir et al., 2009), identified distinct positive and negative countertransference dimensions [e.g., warm/competent, angry/frustrated, aggressive/sexual, failing/incompetent, bored/angry at parents, and overinvested/worried (Satir et al., 2009); warm/attuned, angry/criticized, disorganized/frightened, overinvolved/worried, disengaged/hopeless, and sexualized (Knaus et al., 2016; Tanzilli and Gualco, 2020; Tanzilli et al., 2020)] in therapists of different orientations. Moreover, results have shown that specific countertransference dimensions are predicted by and/or associated with clients’ personality pathology [with clients’ dysregulation and constriction positively predicting therapists’ anger/frustration and negatively predicting therapists’ warmth/competence (Satir et al., 2009; see also Knaus et al., 2016; Tanzilli and Gualco, 2020)], psychological functioning (showing a positive association with positive countertransference and a negative association with negative countertransference; Tanzilli et al., 2020), and therapeutic alliance (positively associated with positive countertransference and negatively associated with negative countertransference; Tanzilli and Gualco, 2020).

Finally, some systematic single and multiple case studies using the Child Psychotherapy Q-Set (Schneider, 2003) in child psychodynamic psychotherapy showed that the client-therapist reciprocal interaction is organized in repetitive patterns of reciprocal interaction (interaction structures) that can be interpreted as positive or negative transference-countertransference matrices (Goodman and Athey-Lloyd, 2011; Ramires et al., 2015; see also Ramires et al., 2020; Odhammar et al., 2019).

### 2.2 Therapeutic techniques

#### 2.2.1 Interpretations

Interpretation is, at least in more traditional approaches, one of the key therapist interventions to promote change in PPCA (Delgado et al., 2015). Traditionally, it aims to bring out the latent meaning of a given material (Laplanche and Pontalis, 1988, p. 227), allowing the children/adolescents to gain an insight into their unconscious conflicts and defense mechanisms. As such, in more classical approaches, interpretation (especially of transference neurosis—see later in this article) is the primary means of achieving insight and thus plays a central role in the PPCA change process. More recently, however, interpretation has been conceptualized as the therapist’s attempts to verbally modulate the steadily co-constructed intersubjective field with the children/adolescents to foster their mentalizing and symbolizing abilities that, in turn, may allow a reorganization of their implicit relational knowing (see a later section in this article) (Delgado et al., 2015; Fonagy and Target, 1998; Muñoz Specht et al., 2016). Terradas and Asselin (2021) suggest that clarifications and confrontations should prepare a child for an interpretation and provide insight into pre- or unconscious content the child has shown through play (see a later section in this article). Subsequently, beyond verbal interpretations, interpretation should also be made during the child’s play activity (joining in on play). Finally, perlaboration (or “working through”; see a previous section in this article) should help the child assimilate an interpretation and overcome the resistance associated with it. This process allows children to gain access to resistance, accept repressed content, and break out of the compulsion to repeat.

Some empirical studies have focused on the role of interpretation in promoting change in PPCA. For example, Fonagy and Moran (1990) showed, through an experimental single-case design, that uncovering and raising awareness of unconscious conflicts through interpretations predicted good outcome. The following paragraphs focus on some specific forms of interpretation in PPCA.

##### 2.2.1.1 Interpretation of transference and countertransference

The interpretation of transference and countertransference plays a relevant role in PPCA, especially because of their focus on the here and now, which makes them preferable to genetic interpretations, especially with children (Delgado et al., 2015). As stated by Wittenberger (2016), patients can project internal objects onto the analyst (p. 118) and thus make experiences of relationships visible. Therapists must allow themselves to become entangled in the transference offered by the patient to make conflicts visible and pave the way for an emotionally corrective experience (Castonguay and Hill, 2012). Therefore, the interpretation of transference and countertransference works by making conflicts visible, empathizing with them, and understanding them (Tyson and Tyson, 1986). More specifically, interpreting transference (especially transference neurosis) allows the analyst to relive and understand the patient’s relational experiences. At the same time, the interpretation of countertransference allows the analyst to empathize on a deep mental level. In this way, the transference process can be understood and mentalized together. Moreover, since the parents or caregivers can also be included in the treatment of children, the transference and countertransference dynamics and related interpretations must also be considered at this level. The difference between transference interpretations in treating children and adults lies in the type of communication. While an interpretation is communicated verbally to adults in the context of their conversation, especially with children, it can also be made in the context of a play episode (see a further section in this paper) through a combination of verbal and non-verbal expressions.

Some studies have been conducted on transference interpretation in PPCA, while the same cannot be said for countertransference interpretations. Luzzi et al. (2015), for example, found through a mixed-methods design that transference interpretations, especially when dealing with the expression of anxiety, were associated with an increase in clients’ symbolic activity and a decrease in acting out during play. In another study, Della-Rosa (2016) explored through thematic analysis the topics on which psychoanalysts make transference interpretations in the treatment of four depressed adolescents. Results showed that these tend to focus on negative/conflicting or difficult-to-express feelings toward the therapist, dependency issues related to resistance or attachment to the therapist, and the desire for more sessions and fear of rejection. However, whether or to what extent these transference interpretations lead to psychotherapeutic change was not investigated. In another study, Della Rosa and Midgley (2017) showed through conversation analysis that when therapists make transference interpretations about the ending of the treatment in short-term therapy, adolescents tend to react in either a dramatizing or down-playing way to deal with the anxiety regarding the separation from the therapist. Jones et al. (2020) conducted a qualitative investigation in the context of a clinical trial aiming at assessing the effects of transference interpretation in psychodynamic therapy for adolescents with depression. Results showed that transference interpretations are experienced as useful for enhancing insight and self-esteem and strengthening the therapeutic relationship while refraining from them can be perceived as limiting by the therapists. However, this might be useful, especially with adolescents with lower levels of functioning. Finally, in a clinical trial where adolescents were randomized to the same therapist working either with or without transference interpretation, it was shown that both treatment conditions were equally effective on overall clients’ functioning. Still, the transference work group was more effective (at treatment end and follow-up) concerning depressive symptomatology (Ulberg et al., 2021).

##### 2.2.1.2 Interpretation of dreams

The interpretation of dreams also plays a role in the PPCA change process. Children’s dreams “show the most urgent concerns and tasks for children in different phases of development. At the same time, dreams illustrate the development of ego functions such as verbalization, language development, competence in dealing with affects and cognition” (Ablon and Mack, 1980, p. 184). For this reason, their interpretation shows how many levels are touched by the child’s dream production and its analysis. Few studies have addressed whether dream interpretation as an intervention is responsible for psychotherapeutic changes. However, individual case reports with corresponding descriptions of dream narratives and their interpretation have a long tradition in psychoanalytic publications emphasizing their relevance (e.g., Ablon and Mack, 1980). Gillman (1987) described the case vignette of an eight-year-old girl with obsessive-compulsive symptoms and pronounced sibling rivalry and concluded that dream interpretation is a largely neglected field in child psychoanalysis but one that offers potential for psychotherapeutic change.

Lempen and Midgley (2006) attempted to examine the role of dream interpretation in psychoanalytic practice in a qualitative study interviewing psychoanalysts about the importance of dream work in their work with children and adolescents. The respondents agreed that children are more likely to bring dreams into treatment when there is a strong psychotherapeutic alliance and that the importance of the dream is crucial for the transference. Colace (2010) conducted four large-scale studies on children’s dreams between 1989 and 1999 to empirically test Freud’s theories on children’s dreams, showing wish fulfillment in children’s dreams.

##### 2.2.1.3 Interpretation of defense mechanisms

Several authors agree on the relevance of the defense interpretation in promoting therapeutic change in PPCA (e.g., Göttken and von Klitzing, 2015). The analysis of defense mechanisms provides information about the patient’s personality structure and previous inner-psychological development. However, empirical studies supporting this view are still few. For example, in a seminal investigation, Truax and Wittmer (1973) showed that the focus on adolescent defense mechanisms was associated with greater progress both at the end of treatment and at the one-year follow-up. Prout et al. (2019) investigated the relationship between resilience, defense mechanisms, and implicit emotional regulation. Consistent with the idea that the focus and verbalization of defense mechanisms would evoke a change in emotion regulation and thus resilience, results showed that the recognition of defense mechanisms and the act of verbalization could lead to symptom reduction and improvement (for a study on defense mechanisms of parents of children with emotional problems, see also Di Giuseppe et al., 2020).

##### 2.2.1.4 Interpretation of resistance

Children can show the same resistance as adults and develop further resistance related to both their stage of development and their dependence on caregivers. Resistance to the goal of treatment provides information about inner psychological conflicts and the patient’s ability to deal with them (e.g., through defense mechanisms). Resistance is not always visible in the work with children but more often in the work with parents (Freud, 2018; Miller, 1993). At a general level, the relevance of resistance interpretation for the change process in PPCA can be found in treatment manuals (Göttken and von Klitzing, 2015; see also Gatta et al., 2019) or individual case studies (e.g., Ramires et al., 2015), but empirical studies explicitly addressing resistance interpretation in PPCA seem to be lacking. More specifically, Danneberg and Eppel (1980) addressed parental defenses and resistance in child psychodynamic psychotherapy, stating that psychotherapists must always be aware of the influence of parents’ resistance on children. Analogously, Windaus (2006) described brief and focal psychodynamic therapy with children, adolescents, and their parents and stressed the importance of addressing parents’ resistance during the therapeutic work. The brevity of the treatment—which is also clear to the patient in advance—can result in a potential for resistance to not engage with the treatment.

#### 2.2.2 Verbalization

Verbalization is an expressive intervention with which the psychotherapist attempts to name conflicts and affects and provide “unspeakable” things with word meanings (Fonagy and Target, 1998). Freud (2018) emphasized that verbalization serves as the first step towards awareness and helps to cope with the secondary process (see also Fonagy and Moran, 1990). However, verbalization is distinguished from interpretation in that it is limited to describing behavior, acting out behavior, or affects and does not add any meaning. There are almost no empirical studies on verbalization in PPCA. In one of these, a systematic single-case study, the authors showed that, in the three-year psychoanalytic process of a teenager who was treated five times a week, the verbalization of conflicts was strongly associated with outcome (Moran and Fonagy, 1987).

#### 2.2.3 Mirroring

Mirroring (i.e., the therapist reflecting on the client’s experience and sharing with them how they might feel) is also firmly anchored in the psychoanalytic literature because the treatment “has in the broadest sense the function of the face, reflecting what is visible.” (Winnicott, 2010, p. 135). Generally, it can be argued that there is a consensus on the function of mirroring in PPCA as a relevant supportive technique aimed at providing empathic validation (Allen and Fonagy, 2006). Terradas and Asselin (2021) describe various goals of mirroring in their work on play with children with early relational trauma (e.g., fostering a sense of security in children, developing children’s mentalizing abilities, symbolizing problems). In addition, mirroring can be used as the psychotherapeutic process develops and the children’s vocabulary expands to name emotions or internal states. In this case, the boundaries between mirroring and verbalizing are fluid.

Despite this relevance, the role of mirroring in PPCA has received little empirical attention, with the exception of a few case studies. For example, a case study by Trowell et al. (2003) examined depressed children and adolescents and their parents and showed that mirroring interventions played an essential role in promoting change, especially in the early phases of treatment.

#### 2.2.4 Free play

From their origins, psychodynamic theories have emphasized the importance of free play in child psychotherapy (Klein, 1955). Free play is a non-directive and non-utilitarian therapeutic technique that leaves it up to the child to decide what to play. The psychoanalyst holds back on instructions and gives the child the lead. As such, it represents a privileged context where children can practice their “as if” skills through a form of symbolic communication, allowing them to acknowledge and express their inner states and psychological processes (e.g., feelings, anxieties, conflicts, defenses, desires) within a shared relational context. At the same time, it can be seen as a kind of equivalent of free association in the psychodynamic psychotherapy of adults (Porter et al., 2009).

Beyond the several clinical case descriptions focused on free play in PPCA (e.g., Kernberg, 2006), there are also some empirical investigations in this regard. For example, Leudar et al. (2008) used an ethnomethodological case study to investigate four consecutive psychoanalytic group sessions with children. Their findings indicate that a change-promoting psychotherapeutic process arises through verbalizing issues that become visible through free play and interaction. Analogous results were reported by Carlberg (2009) in a study of the psychodynamic child psychotherapy change process through quantitative and qualitative methods. Significant clinical changes were observed in most of the children, and free play played an important role in these changes, allowing the children to organize and symbolically express their inner experiences in new ways. With this regard, the author emphasized the importance of play interaction and the psychotherapist’s ability to create affective engagement in the play situation.

Halfon and Bulut (2017) investigated the relationship between symbolic play, affect regulation, and within-session adherence to mentalizing principles in children with behavioral disorders who received psychodynamic play psychotherapy. The authors found that an increase in symbolic play offers the context for the development of affect regulation when the therapeutic work is organized coherently with the prototype of a session that promotes mentalization (rated by independent judges) (see also Halfon et al., 2021). Furthermore, some other multiple systematic single-case studies on psychodynamic play therapy showed that play offers children the possibility to express a variety of inner psychological states associated with their presenting problems; the play profiles reflect a continuum of coping strategies varying from less to more adaptive; throughout the treatment, children nonlinearly oscillate between these different play profiles; and a positive outcome may require the destabilization of less adaptive play profiles and an increase in the variability of the actual play profiles so that qualitatively new and more adaptive coping strategies can emerge (Halfon et al., 2016; Halfon et al., 2019b).

## 3 Common factors in PPCA

In the present section, common factors of PPCA are organized into relational, patient, therapist, and parent and interpersonal environment factors. Moreover, we address mentalizing and play as common factors involving both clients and therapists (and eventually, the parents) (see Table 2).

**Table 2:** Common factors of PPCA.

Domain	Therapeutic factor
Relational factors	Therapeutic alliance Internal representation of the therapeutic relationship/interaction structures
Patient factors	Active seeking of help/willingness to participate Motivation/readiness for change Treatment involvement Positive expectations and hope
Psychotherapist factors	Interpersonal skills (empathy, warmth, and genuineness) Direct influence skills (providing an active structure to the session, a rationale for the treatment, and instructions and guidance) Credibility Involving parents and triangulating Playing ability Flexibility Treatment expectations/Allegiance
Parent and interpersonal environment factors	Parental willingness to participate Parental treatment involvement Parental Perceived barriers to treatment participation and therapeutic change Parental treatment expectations Family dynamics Parent-therapist alliance Social support
Mentalizing	Therapist mentalizing Patient mentalizing Parent mentalizing
Play	Symbolization Affect regulation Mental state talk Patterns of interaction

### 3.1 Relational factors

It is assumed that relational factors play an important role in psychotherapy (Wampold and Imel, 2015). The therapeutic alliance and related factors have been shown to significantly predict treatment outcome in adult psychotherapy (e.g., Flückiger et al., 2018). A similar argument can be made for psychotherapy with children and adolescents. For example, Kernberg et al. (2012) proposed recommendations for child psychotherapy, some of which deal with the *therapeutic alliance*. These emphasize that in order to create a safe psychotherapeutic space and promote appropriate alliance-building, therapists should carefully consider the child’s developmental level and personality structure within a setting that ensures respect for the child’s autonomy. A shared understanding of the child’s behavior/symptoms should also be developed with parents or caregivers. Analogously, it has been stated that the work with adolescents poses particular challenges in the adequate building of the therapeutic alliance because these latter, because of their developmental stage, tend to minimize psychological problems (Shirk and Saiz, 1992) and mistrust adult authority (DiGiuseppe et al., 1996), thus complicating the engagement in the therapeutic relationship.

In a qualitative study of children’s experiences of their therapeutic relationship, Baylis et al. (2011) developed an empirically informed process model of the psychotherapeutic alliance with children (Child Alliance Process Theory). Such a model posits that the therapeutic alliance with children develops in layers (for an example in adult psychotherapy, see Gelo et al., 2016). First, therapists engage the child in the relationship by proposing activities, expressing care, listening actively, and attending to ruptures. Then, therapists strengthen the building alliance by further proposing activities, demonstrating patience, validating feelings, and respecting privacy and confidentiality. These findings appear to be supported by another qualitative study that longitudinally examined the child-therapist and parent-therapist relationships as agents of change from the perspectives of children, parents, and therapists (Núñez et al., 2022). The results showed that the therapeutic relationship in the early sessions facilitated change by allowing children’s reticence to be addressed, thereby improving their positive attitudes toward therapy; supporting children’s intrapersonal changes through the opportunity for positive interactions; and increasing parents’ confidence in the therapist’s ability to overcome the challenges initially presented by the child. However, in later stages of treatment, the therapeutic relationship facilitated change by allowing the children to become increasingly involved in the therapeutic process and to feel accepted and free by the therapist, leading the children to feel that the therapist helped them to change; facilitated cooperative engagement of therapists and parents, for the former through a flexible and child-centered attitude, for the latter through the therapist’s ability to establish an affective relationship with the children.

Concerning quantitative research, meta-analyses support a relevant (small to medium) effect of the therapeutic alliance on outcome in psychotherapy for children and adolescents, including PPCA (Karver et al., 2006, 2018; Shirk and Karver, 2003; Shirk et al., 2011). At a more specific level, Halfon et al. (2019a) found that the therapeutic alliance in child psychodynamic therapy has a high-low-high time course, with children with internalizing and externalizing problems showing a decreasing and increasing alliance curve, respectively. In addition, the time course of the overall alliance predicted treatment outcome. Halfon (2021) showed that psychodynamic techniques in psychodynamic child psychotherapy best predicted treatment outcome when used in the context of a high therapeutic alliance. Similar results were found by Ramires et al. (2022).

Other empirical studies examined specific elements of the psychotherapeutic relationship and their association with treatment outcome in PPCA. Atzil-Slonim et al. (2015) showed that adolescents developed both positive and negative *internal representations of their therapeutic relationship* over the treatment, with an increase in the former and a decrease in the latter being associated with greater treatment satisfaction. Moreover, the development of these relational representations was associated with an improved perception of the relationship with parents. These findings are particularly significant in that they support the idea that in PPCA, work in the here and now can facilitate the development of new interpersonal representations of the therapist in the client, which in turn affect other important relationships (Atzil-Slonim et al., 2015). In another study, Halfon et al. (2018) identified four *interaction structures* in psychodynamic child psychotherapy using the Child Psychotherapy Q-Set: therapeutic alliance, children’s emotional expression, child-centered techniques, and psychodynamic technique. Although the therapeutic alliance factor explained the highest variance in the data set, it was not found to predict outcome (along with children’s emotional expression).

Some studies have focused on the contribution of pre-treatment characteristics or in-session behaviors to alliance development in therapeutic approaches other than psychodynamic. Regarding the former, for example, it has been found that adolescent maltreatment experiences and severity of interpersonal problems predict, respectively, difficulties in initial alliance formation and alliance development and, consequently, poorer outcome (Eltz et al., 1995; see also Colson et al., 1991). Regarding the latter, some studies have shown that the development of the therapeutic alliance is associated with therapists paying attention to the youths’ personal experience, setting meaningful goals, and presenting themselves as allies in adolescent family therapy (Diamond et al., 1999), the use of a collaborative language in child cognitive-behavioral therapy (Creed and Kendall, 2005). Moreover, Russell et al. (2008) found that the development over time of therapists’ responsivity, experiential socialization, and remoralization predict subsequent therapist and client-rated alliance in cognitive-behavioral therapy for adolescents. Although promising, these findings suggest that the role of the therapeutic alliance and other relational factors in the change process of PPCA needs to be further examined in the future.

### 3.2 Patients’ factors

The general literature on common factors often mentions the following patient factors: active help-seeking and willingness to participate, motivation and readiness for change, therapy involvement, positive expectations, hope, and belief in treatment (e.g., Tracey et al., 2003). These factors also seem to play a relevant role in the psychodynamic treatment of children and adolescents. *Active seeking of help* and *willingness to participate* play a relevant role in PPCA, as shown by a meta-analysis of Karver et al. (2006) reporting medium effects of willingness to participate on treatment outcome. This is further related to children and adolescents’ *motivation* and *readiness for change* as important factors in PPCA. Some empirical research has been conducted in this regard, showing that young people with higher motivation and commitment to therapy develop better therapeutic alliance (Ellis et al., 2012; Estrada and Russell, 1999; Fitzpatrick and Irannejad, 2008) and may have better outcomes (Black and Chung, 2014; Adelman et al., 1984; for contrary results, see Killips et al., 2012). The same can be said concerning children and adolescents’ *treatment involvement*, as shown by a meta-analysis of Karver et al. (2006), which found a moderate effect of actual participation in therapy on treatment outcome.

*Positive expectations* and *hope* of children and adolescents are given relatively little attention in the literature on PPCA and are also not reflected in treatment manuals. However, its relevance has been emphasized by the development of the Hopes and Expectations for Treatment Approach (HETA; Urwin, 2009) as an assessment tool to evaluate child psychotherapy’s effectiveness. In a mixed-methods investigation of the expectations and experiences of children treated with psychodynamic psychotherapy, Carlberg (2009) found that the majority of the children expressed positive expectations and hopes regarding the treatment before it started and, after termination, considered their therapy experience as positive or very positive. However, a survey of young clients by Watsford and Rickwood (2014) did not find a relationship between their initial expectation and treatment outcome, even though the latter was predicted by clients’ experience of therapy and their preference for personal engagement in therapy. This area certainly needs further research. Despite these promising results, there is a need for further empirical research on the role of patient factors in the change process of children and adolescents’ psychotherapy change processes.

### 3.3 Psychotherapists’ factors

Sprenkle et al. (2013) underline that the person of the psychotherapist plays a fundamental role in the psychotherapeutic alliance: Therapists differ as people not just in what they do but also in who they are. Clients react to therapists as therapists but also as people. A therapist has an age, a gender, a culture, a way of speaking and being. “(p. 92). With this regard, so-called interpersonal skills (i.e., empathy, warmth, and genuineness) are considered to play a pivotal role in psychotherapy, especially in relation to the possibility of building and managing an adequate therapeutic alliance (e.g., see Grencavage and Norcross, 1990; Tracey et al., 2003). The therapist’s intersubjective ability to recognize their own and others’ internal states and to understand the development of the relationship between them and the patients are of great importance in this respect (e.g., see Grencavage and Norcross, 1990; Tracey et al., 2003).

These factors may also be relevant in PPCA. In one seminal study investigating therapists’ *interpersonal skills* as predictors of outcome in child therapy, Truax et al. (1973) showed that high therapist’s empathy, warmth, and genuineness produced greater behavior and personality change, while low levels of these variables produced even a worsening of the children condition. More than 30 years later, the meta-analysis conducted by Karver et al. (2006) produced analogous results. The same meta-analysis showed that therapists’ *direct influence skills* (e.g., providing an active structure to the session, a rationale for the treatment, and instructions and guidance) also positively impact treatment outcome (Karver et al., 2006).

Such influence skills have also been shown to enhance therapist *credibility* (i.e., the extent to which they are perceived attractive, trustworthy, and having expertise) (Hoyt, 1996). Therapist credibility is supposed to instill positive expectations, hope, and faith in the client (Frank and Frank, 1991), thus positively influencing treatment process and outcome (for a meta-analysis on adult psychotherapy, see Hoyt, 1996). However, although there have been some empirical investigations exists in children and adolescents psychotherapy (e.g., Stein et al., 2001), more empirical research is needed.

The following therapeutic factors also play a relevant role in the treatment of children and adolescents. First, the *ability* to *involve* and *triangulate with parents*, that is, the ability to build and manage different relationships with the patient’s family members and to analyze how these relate to the client’s relationship patterns with the therapist (Hanley and Noble, 2017; for a review, see Ruberman, 2009). The modality and frequency of contacts may depend on the patient’s developmental stage and is crucial, especially for children. From the beginning, the therapist must make explicit to the child or adolescent the nature and meaning of this parental involvement in a shared, clear, and transparent way (Kernberg, 2006). Such involvement has the primary function of gaining parental cooperation by clarifying treatment goals and strategies, which in turn is essential for establishing and maintaining a therapeutic setting (e.g., Kehr and Köpp, 2018). It may also be useful in gathering information about specific events concerning the patient or more general family dynamics, which in turn may be useful in therapeutic work with the child/adolescent. In addition, the therapist may provide psychoeducational support to parents, for example, by trying to facilitate their understanding of the patient’s situation and/or promoting parenting skills (e.g., Martinez et al., 2017). The therapist can provide emotional containment to parents in times of particular need. Second, the therapist’s *ability to play*. Psychotherapists who work with children must be able to engage and be engaged in a game or allow themselves to be used as an object in the game (Kernberg et al., 2012; see the later section on play in this article). Third, flexibility, which in working with children and adolescents refers to the ability to evaluate the nature and timing of interventions according to the patient’s developmental stage (Karver et al., 2005). A final therapist factor we would like to address is *allegiance* (i.e., the extent to which the therapist believes the therapy is effective) (Wampold and Imel, 2015), which has been suggested as a common factor that plays a relevant role in determining treatment effectiveness. Unfortunately, the authors could not locate any empirical research on these therapist factors.

### 3.4 Parent and interpersonal environment factors

Parents’ participation, expectations, and motivations positively influence treatment outcome (Karver et al., 2005). The importance of involving parents is illustrated, for example, by a meta-analysis showing that both parents’ *willingness to participate in treatment* predict therapy outcome (Karver et al., 2006). More specifically, it has been found that the severity of children’s behavioral problems and degree of parental distress reduces the within-session *involvement* of parents (Haine-Schlagel et al., 2012), which in turn can be promoted by in-session parental psychoeducation (e.g., discussing the child problem causes and providing a treatment rationale) (Martinez et al., 2017). In addition, Kazdin and Wassell (2000) found that the extent to which parents *perceived barriers to treatment participation and therapeutic change* was associated with their perceptions of treatment relevance and outcome. Further, the perception of both treatment barriers and change was predicted by parental psychopathology and lower levels of quality of life (see also Fonagy and Target, 1995).

Nock and Kazdin (2001) investigated *parental expectations* of therapy, finding that these predict treatment barriers, attendance, and premature termination. With this regard, Shuman and Shapiro (2002) showed that providing parents with preliminary information about psychotherapy increases the accuracy of their treatment expectations and, at least to a minimal extent, treatment attendance.

In the treatment of children and adolescents, *family dynamics* (e.g., intergenerational conflicts, family role allocation, parenting practices, interplay between attachment styles, parental relationship experiences, ego strengths, and triangulation skills of the parents as well as their introspection and interpersonal) may also play a relevant role (e.g., Möhring, 1999). There has been no specific empirical research on this in psychotherapy with children and adolescents, except for parenting practices (see Kennedy and Midgley, 2007; Ng et al., 2021).

Another important common factor regards the *parent-therapist therapeutic alliance*, which, analogously to the client-therapist alliance, involves an emotional bond and agreement on goals and tasks between the parents and the therapist (see Accurso et al., 2013). Two qualitative studies by Núñez et al. (2021, 2022) investigated how the triadic (parents, child, and therapist) dynamic nature of the therapeutic relationship influences the change process. The results indicated that the therapist’s commitment and playful stance, as well as the parents’ close and adaptable attitude, collaboration, and mutual involvement between child and therapist within a caring, validating, and trustful relationship, influenced the parents’ and children’s motivation and facilitated change and the development of socio-affective tools.

One of the first meta-analyses on parent-therapist alliance estimated a relatively small effect on patient change (Karver et al., 2006). Analogous results were produced by a later meta-analysis by McLeod (2011). In line with this, other empirical studies suggest that while the child-therapist alliance seems to be more associated with client change, the parent-therapist alliance seems more associated with treatment continuation and lower levels of dropout (Kazdin et al., 1997; Weisz et al., 2005; Zack et al., 2007 see also Hawley and Weisz, 2005).

Finally, regarding the broader interpersonal environment of the patient, it has been shown that *social support* predicts higher patient-therapist therapeutic alliance (Shirk and Karver, 2003) and treatment outcome (Black and Chung, 2014). Analogously, interpersonal problems in social relationships predicted difficulties in establishing an adequate therapeutic relationship (Eltz et al., 1995).

### 3.5 Mentalizing

Mentalizing (or reflective function; RF) can be defined as the metacognitive ability „to understand others’ and one’s behavior in terms of mental states “(Fonagy and Allison, 2014, p. 1). It has been shown that its development fundamentally contributes to adaptive socio-emotional-cognitive outcomes (Schore, 2014), while a lack of mentalizing is related to several psychopathological conditions (Bateman and Fonagy, 2012; for a social constructionist account of psychopathology, see Gelo et al., 2015b). Although mentalizing has also been conceptualized as a treatment outcome, an increasing number of scholars suggest it represents a therapeutic factor (for an empirical review, see Katznelson, 2014). Moreover, although this concept originates within the psychodynamic tradition and is explicitly implemented in mentalization-based treatments for adults (Bateman, 2022) and children (Lindqvist et al., 2023), the relevance of such a construct as a common therapeutic factor is emphasized by an increasing amount of scholars (Bateman et al., 2018; Fonagy and Allison, 2014; Montgomery-Graham, 2016). As stated by Goodman et al. (2016), “If this hypothesis is valid, then various treatment models are effective because they revive the patient’s capacity to interpret behavior as motivated by the underlying mental states of self and other” (p. 3; see also Bateman and Fonagy, 2017), with different models enhancing/focusing on various dimensions mentalizations (self/other, implicit/explicit, affective/cognitive).

From this perspective, it has been suggested that it is fundamental for child and adolescent therapists to adopt a *mentalizing attitude* or stance (also called RF approach) (Fonagy and Adshead, 2012). Muñoz Specht et al. (2016) developed a conceptual framework of mentalization-based interventions through a qualitative study of the sessions of two experienced child and adolescent psychodynamic psychotherapists. An overall mentalizing stance principle emerged, which entailed the “therapist’s orientation toward the patient’s inner world” (p. 289). This comprised interventions aimed at promoting the patient’s exploration and emotional expressions of their inner states and the development of alternative perspectives regarding the self, the other, and their relationship within an empathic, caring, supportive, and self-reflecting relationship both within and outside the play context. Some studies have been conducted on the therapist RF approach in child and adolescent psychotherapy, showing its association with therapeutic alliance, symbolic play, and affect regulation (Halfon and Bulut, 2017; Halfon et al., 2017b; Ramires et al., 2022) as well as with treatment outcome (Halfon et al., 2019b).

Analogous results have been found concerning the *patient’s mentalizing abilities*. For example, Ramires et al. (2022) found that increased children’s RF abilities positively impact patient-therapist interaction structures. Similarly, it was shown that while lower baseline levels of children’s RF correlate with more problematic behaviors, an increase in children’s RF is associated with treatment outcome (Oehlman Forbes et al., 2021). Finally, it can be argued that also *parents’ mentalizing* can be a relevant factor in child and adolescent psychotherapy. For example, Halfon and Besiroglu (2021) found that parents’ RF predicted a reduction in children’s problematic behavior over treatment. Although most of the studies indicated above (these studies) have been conducted in the context of psychodynamic approaches, research employing the Child Psychotherapy Q-set (Schneider, 2003) has shown that a focus on mentalization characterizes not only PPCA (e.g., Carvalho et al., 2019; Di Lorenzo et al., 2015) but also other child and adolescents’ psychotherapeutic approaches [e.g., cognitive-behavioral (Goodman et al., 2016) and child-centered (Prout et al., 2018) child therapy]. Overall, the existing results are promising. However, further empirical research is needed to assess better the role of mentalizing in child and adolescent psychotherapy.

### 3.6 Play

Although the relevance of play in the therapeutic process of child psychotherapy has been originally emphasized in the psychodynamic tradition (see a previous section in the present paper), several other approaches have been increasingly acknowledging the central role of play in promoting change in child psychotherapy (Porter et al., 2009). Notwithstanding the existing differences, it is possible to identify common features characterizing play’s therapeutic role in child psychotherapy. For example, Schaefer and Drewes (2013) developed a transtheoretical play therapy model inspired by humanistic, psychodynamic, and cognitive-behavioral therapy. Such a model identifies 20 therapeutic core agents of play differently related to cognitive, emotional, and interpersonal processes and considered to facilitate communication, foster emotional wellness, and increase personal strengths. As stated by the authors, “play actually helps produce the change and is not just a medium for applying other change agents nor does it just moderate the strength or direction of therapeutic change” (Schaefer and Drewes, 2013, p. 2).

Such a view is consistent with the idea that symbolic play is a facilitator for the development of the child’s ability to mentalize, providing a safe environment for exploring different aspects of one’s own experience and fostering the child’s ability to imagine the inner experiences of play characters, their thoughts, beliefs, intentions, and feelings (Fonagy and Target, 1995; for an account of the developmental and clinical functions of safety and intersubjective mentalizing, see Podolan and Gelo, 2023, 2024). In this context, the play offers the therapist a privileged frame to facilitate the child’s *symbolic abilities* by identifying the underlying mental states of their behavior during play or verbalizing the desires or intentions of the characters involved in the play and their connections to significant other in the child’s life, such as the parents (Zevalkink et al., 2012). During play, the use of mental discourse by therapists in relation to children’s mental states allows these latter to organize their own experiences so that they have a better understanding of their inner world (Fonagy and Target, 1998); this, in turn, can help them to formulate hypotheses about the link between emotions and behaviors, and then develop the ability to communicate and regulate emotions (Fonagy and Target, 1998).

Several meta-analyses support the effectiveness of play in child psychotherapy (e.g., Drisko et al., 2020). However, only a few empirical investigations have focused on the play process. Beyond some qualitative single-case studies providing preliminary evidence about the relevance of the play process for healing and therapeutic change in child psychotherapy (e.g., Campbell and Knoetze, 2010; Ebenstiner, 1998), quantitative research employing the CPTI (Kernberg et al., 1998) has shown that an increase in children’s symbolic play is associated with or predict better *affect regulation* (e.g., Halfon and Bulut, 2017; for systematic case studies, see Chari et al., 2013; Chazan and Wolf, 2002), which in turn predict positive treatment outcome (Halfon et al., 2019b) in psychodynamic child psychotherapy. Moreover, Halfon et al. (2017b) found that play offers children the possibility for *mental state talk* (i.e., words used to attribute inner states to others), and when parents are involved in the play activity, the child’s role-playing ability is associated with both children and mothers’ (but not fathers’) play-related mental state talk. Moreover, there was an association between the child’s affect regulation in play and the mother’s mental state talk, while the ability of children and mothers to verbalize mental states during pretend play was associated with less internalizing problems. On the contrary, out of pretend play, the mental state talks of children, mothers, and fathers were associated with more externalizing problems (see also Halfon et al., 2017a).

A series of systematic case studies employing the Child Psychotherapy Q-Set (CPA; Schneider, 2003) have examined the role of symbolic play in psychodynamic child psychotherapy. Findings showed that symbolic play is positively associated with the therapist’s mentalizing stance and the therapeutic alliance (Ramires et al., 2022) and that play-related processes (e.g., children engaging therapists in play, therapists trying to understand children’s play) are among the characteristic issues of children’s repetitive patterns of interaction (Ramires et al., 2015; see also Ramires et al., 2020). Although these studies were conducted in the context of psychodynamic child therapy, they highlight the extent to which play and related processes can contribute to the process of change in child psychotherapy in general.

## 4 Discussion and conclusion

One of the aims of psychotherapy science is to identify the therapeutic factors that make a given treatment effective (Gelo and Manzo, 2015; Gelo et al., 2015a; Gennaro et al., 2019). In this paper, we described several specific and common therapeutic factors that have been postulated in PPCA. On the one hand, most of them resonate with therapeutic factors described in adult (psychodynamic) psychotherapy, although specifically declined for children and adolescents. This is the case for school-specific factors regarding clinical processes (i.e., insight, working through, remembering and reconstructing, catharsis, abreaction and regression, transference and countertransference) and therapeutic interventions (i.e., interpretation, verbalization, and mirroring) as well as for common factors regarding relational factors (e.g., client-therapist alliance), patient factors (e.g., motivation, treatment involvement, expectation and hope), therapist’s factors (e.g., interpersonal skills, allegiance), and therapists’ and clients’ mentalizing. On the other hand, some of these specific and common factors appear to be unique to psychotherapy for children and adolescents. This is the case, for example, for the relevance of the play process (which can be considered specific for PPCA when free or common to other approaches when more structured) and the school-independent therapist’s ability to facilitate play when working with children in both PPCA and other therapeutic approaches. Other examples include the common factors of parents’ involvement in the therapeutic process (including their willingness to participate, motivation, and alliance with the therapist) and their mentalizing abilities.

These common and specific factors, most of which align with those identified by Kazdin et al. (1990), refer to technical, relational, and personal aspects and related clinical processes that steadily interact with each other and can only promote change because of that interaction. Therapists’ interventions—which can be specific for PPCA (e.g., interpretation, verbalization, mirroring, free play) or common to other approaches (e.g., play)—are intended to trigger change-promoting clinical processes—both specific to PPCA (e.g., insight and working through, remembering and reconstructing, catharsis, transference/countertransference) and common to other approaches (e.g., mentalizing factors)—in interaction with a relational context characterized by a good therapeutic alliance among the actors involved and by adequate levels of personal factors (e.g., clients’ and parents’ expectations, motivation, and involvement) that are common to different approaches. Such an account is coherent with current trends within the specific-common factors debate endorsing to move from an “either/or” to a “both/and” position. According to them, psychotherapy works because of a given interaction and synergy of specific and common factors. As stated by McAleavey and Castonguay (2015), “common and unique factors most likely work symbiotically (and sometimes parasitically) with one another” (p. 294), such that “a selective combination of common and specific factors should be employed in the treatment of each client.” (Lampropoulos, 2000, p. 288; for empirical evidence, see, e.g., de Felice et al., 2019; Tschacher et al., 2014).

Further research is needed to empirically assess the role of specific and common therapeutic factors in PPCA (for a methodological review, see Gelo et al., 2009, 2020a, 2020b). In fact, except for the therapeutic alliance and, to a lesser extent, some patient and therapist factors, most of the specific and common therapeutic factors reviewed lack any empirical support or present minimal support (mainly through anecdotal case studies and, in some cases, systematic single-case studies). This is especially the case for some clinical processes (e.g., insight, working through, remembering and reconstructing, catharsis, abreaction, and regression), therapeutic techniques [e.g., interpretation, (free) play], and parent and interpersonal environment factors (e.g., parental motivation, involvement and expectations, and social support). In addition, most of the empirical studies reviewed focused on psychotherapy for children, whereas the literature seems to have paid less attention to the empirical investigation of the therapeutic factors of psychotherapy for adolescents. Finally, it should be noted that we considered mirroring and free play as specific therapeutic techniques of PPCA, although these are used also in other approaches (e.g., in child-centered play therapy; for a review, see Porter et al., 2009). This was done because of their historical origin in the context of psychodynamic psychotherapy.

In the future, systematic reviews should more thoroughly assess the empirical support for different specific and common factors in PPCA. Moreover, more primary empirical studies should be conducted, especially on the therapeutic factors of psychodynamic psychotherapy for adolescents. This would provide more empirical support for the latter and for the distinction between the therapeutic factors of psychodynamic psychotherapy for, respectively, children and adolescents. In addition, future research should more clearly assess the extent to which mirroring and free play can be considered therapeutic factors specific to psychodynamic psychotherapy or common to other approaches (e.g., child-centered play therapy). Qualitative research should better explore participants (i.e., therapists, clients, and parents) subjective experiences and representations about what makes PPCA effective. This would provide empirically informed hypotheses on specific and common factors of PPCA, which could be further tested through quantitative methods. To this aim, adequately complex longitudinal models (e.g., Di Blasi et al., 2022) should be employed within process and process-outcome studies. A specific focus should be on the possible interaction between specific and common factors (e.g., Halfon, 2021). Mediation analyses (e.g., Gullo et al., 2023) would be required to effectively test causal relationships between patterns of interaction of specific and common factors and treatment outcome in PPCA. All these studies should be carried out trying to account for the different perspectives of the involved actors (i.e., therapists, clients, and parents), converge on a set of core instruments considered adequate for the assessment of the relevant constructs (for a review, see Tsiantis and Trowell, 2010), and take into account developmental differences between children and adolescents in testing hypotheses about the mechanisms of change of psychodynamic psychotherapy. Moreover, future studies should attempt to test hypotheses on the similarities and differences of specific and common factors of psychodynamic psychotherapy for children and adolescents on one side and adults on the other. It would also be important to assess the satisfaction of children, adolescents, and their families with treatment (Garland et al., 2007; see also Ciavolino et al., 2020). Finally, meta-analyses should be conducted to provide a summary of the evidence.

The identification of empirically supported specific and common factors in PPCA might increase practitioners’ self-awareness and critical reflection on what makes their work effective. This, in turn, might increase the constructive dialogue and exchange among practitioners of different orientations, which has been considered advisable in the field (Gelo and Pritz, 2020; McLeod, 2017). Finally, psychotherapy training programs in PPCAs should consider these empirically supported therapeutic factors to promote the development of trainees’ basic therapeutic skills and evaluate the impact of these factors on trainees’ development (e.g., Messina et al., 2018).
